# The threads that tie protein-folding diseases

**DOI:** 10.1242/dmm.014985

**Published:** 2014-01

**Authors:** Jeffrey L. Brodsky

**Affiliations:** Department of Biological Sciences, A320 Langley Hall, University of Pittsburgh, Pittsburgh, PA 15260, USA.

**Keywords:** Chaperones, Neurodegeneration, Protein folding

## Abstract

From unicellular organisms to humans, cells have evolved elegant systems to facilitate careful folding of proteins and the maintenance of protein homeostasis. Key modulators of protein homeostasis include a large, conserved family of proteins known as molecular chaperones, which augment the folding of nascent polypeptides and temper adverse consequences of cellular stress. However, errors in protein folding can still occur, resulting in the accumulation of misfolded proteins that strain cellular quality-control systems. In some cases, misfolded proteins can be targeted for degradation by the proteasome or via autophagy. Nevertheless, protein misfolding is a feature of many complex, genetically and clinically pleiotropic diseases, including neurodegenerative disorders and cancer. In recent years, substantial progress has been made in unraveling the complexity of protein folding using model systems, and we are now closer to being able to diagnose and treat the growing number of protein-folding diseases. To showcase some of these important recent advances, and also to inspire discussion on approaches to tackle unanswered questions, *Disease Models & Mechanisms* (DMM) presents a special collection of reviews from researchers at the cutting-edge of the field.

## Launching the DMM Protein-Folding Disease series

The completion of the human genome sequencing project in 2003 provided the research community with a wealth of information on gene organization and genetic regulatory sites, enhancing our understanding of the evolutionary history of our genomes, the varied processes that constitute human metabolism, and the relationship between gene function and specific diseases. Nevertheless, the identification of a locus that is mutated in a specific disease, or of a base that is altered in an open reading frame, serves only as a starting point to uncover why a disease arises. Ultimately, one must understand how the mutation affects the function, or even the folding, of a specific protein.

The notion that a disease can arise from protein misfolding is not new, and a plethora of so-called protein-folding diseases has been identified. The concept that protein conformational disorders might share related properties first emerged from an in-depth analysis of select, mutated proteins that exhibit altered structures and result in distinct pathologies (Carrell and Lomas, 1997). The acquisition of an illegitimate conformation can trigger disease caused by various anomalies, including alterations in stability, solubility, interactions with protein partners, post-translational modification or subcellular residence. Some mutations are ‘silent’ in terms of effects on protein function; however, they can change the protein’s translation rate, which in turn alters conformation and function (Kimchi-Sarfaty et al., 2007). Thus, apparently inert polymorphisms in a gene might affect protein conformation. And, although the effects might be subtle, a phenotype could be exacerbated when other mutant proteins are expressed, or when cells are exposed to stress, or as an organism ages (Jarosz et al., 2010). In light of our growing understanding of the human genome and the emergence of new and improved methods in biochemistry and biophysics, the time is ripe to define how an aberrant conformation leads to disease, and how disease presentation is – in turn – modified by changes in protein homeostasis, or ‘proteostasis’ (Balch et al., 2008).

To this end, we launch a review series entitled ‘Protein-Folding Diseases: Models & Mechanisms’ in this issue of *Disease Models & Mechanisms* (DMM). The series, which will be published across several issues of the journal, includes Review articles, A Model for Life interview, a Clinical Puzzle (a review written by a clinician to describe a disease that is relatively understudied) and an At a Glance poster article. Collectively, these articles represent the perspectives of scientists at the cutting-edge of the field.

We start with a poster article from Julie Valastyan and Susan Lindquist that provides a general overview of the classes and causes of diseases that arise from protein misfolding (Valastyan and Lindquist, 2014). These include loss-of-function diseases, such as cystic fibrosis, antitrypsin deficiency and Gaucher’s disease, each of which arise from defects in the folding of secreted proteins, and gain-of-function diseases, such as Alzheimer’s, Parkinson’s and Huntington’s disease, and some forms of cancer. In these latter cases, the protein can form a toxic conformation as a result of defective folding, which negatively impacts cellular health.

Also in this issue, we present A Model for Life interview with Rick Morimoto, who discusses the serendipity in his early career that ultimately led to seminal work on the cloning of heat shock proteins and the analysis of heat shock factor, a transcription factor that serves as a master regulator of the heat shock response (Morimoto, 2014). These early efforts set the stage for Morimoto’s analysis of the heat shock response and the relationship between this response, cellular proteostasis and human disease.

In one of the three Review articles published in this issue, Walker Jackson outlines the fascinating question of why only one cell type is adversely affected when an aggregation-prone protein is expressed in various cells types in the brain (Jackson, 2014). It is also unclear how different diseases arise from the genesis of a single aggregation-prone polypeptide, the prion protein (PrP). In fact, the clinical outcomes, affected regions in the brain and the pathology associated with PrP-induced damaged can be radically different. These data are in line with the notion that the proteostasis network differs in distinct cell types and that the activity of the networks is age-related. It is also possible that the conformation(s) adopted by PrP give rise to a range of pathologies. In a related article coming up later in the series, Cindy Voisine and Marc Brehme will discuss why cellular chaperone systems might be unable to keep pace with the concentration of damaged proteins that arise during aging. Because the level of protein damage rises over time, an accompanying decrease in chaperone levels or function might exacerbate the onset of cell death. An appreciation of these phenomena is crucial to guide the development of therapeutics to modulate chaperones and temper the effects of cellular stress.

Also in this issue, a Review from Carmen Nussbaum-Krammer and Rick Morimoto outlines how the nematode *C. elegans* has been employed to uncover a phenomenon that is pertinent to several neurodegenerative disorders (Nussbaum-Krammer and Morimoto, 2014). For a long time, it was assumed that cells (particularly neuronal cells) harboring misfolded proteins and aggregates are the only cells affected, i.e. the consequences of misfolded protein accumulation and proteostasis collapse were considered to be cell autonomous. However, studies in the worm model indicate that the health of cells neighboring and even quite distant from cells that accumulate misfolded proteins is affected. Indeed, non-cell-autonomous behavior explains several phenotypes associated with neurological diseases. In the future, it will be exciting to determine whether this phenomenon is evident in other diseases.

In the third Review published in this inaugural wave of the series, Ana Oromendia and Angelika Amon detail the fascinating interplay between aneuploidy, i.e. an imbalance in chromosome copy number, and protein quality-control pathways and aging (Oromendia and Amon, 2014). Aneuploidy decreases cellular and organismal fitness, but more recently it has become apparent that chromosome imbalance contributes to age-associated disorders and cancer. These events most likely arise as a result of protein imbalance, which is a downstream effect of aneuploidy.

Later in the series, we will present a Clinical Puzzle in which David Pulmutter outlines why an aggregation-prone secreted protein, the Z variant of α1-antitrypsin, accumulates in the endoplasmic reticulum (ER) of hepatocytes. The aggregation of antitrypsin leads to liver disease, but emerging data have outlined cellular quality-control systems that might be therapeutically modulated. As hoped, recent work led to the isolation of a drug that targeted one of these systems and corrected disease phenotypes associated with antitrypsin aggregation in a mouse model (Hidvegi et al., 2010). In addition, two Review articles from the research groups of Harm Kampinga and Jason Young will describe how molecular chaperones affect a variety of protein-folding diseases. Kampinga and colleagues list the ‘chaperonopathies’, a group of diverse diseases that arise as a result of specific mutations in different chaperone classes and isoforms. The authors then present and discuss the ‘chaperone barcode’, which summarizes which cellular chaperones are linked to specific disorders. The article from Young focuses more specifically on the action of the ubiquitous Hsp/Hsc70 system, and the role this abundant chaperone plays in the etiology of conformational diseases associated with ion-channel defects. The diseases discussed include cystic fibrosis, which arises from mutations in the cystic fibrosis transmembrane conductance regulator; a form of heart disease that results from mutations in the hERG/Kv11.1 potassium channel; a type of hereditary deafness, which comes about from mutations in the KCNQ4 voltage-gated potassium channel; and a salt-wasting disease that transpires when the function of an epithelial sodium channel, ENaC, is altered. In each case, the role of Hsp70 is complex because the chaperone might be required to fold a protein substrate and/or target it for degradation.

Another article, from Sean Ferris and Randy Kaufman, will describe a class of conformational diseases that arise because they are linked to a single cellular compartment. Specifically, the authors outline the myriad diseases that appear due to the misfolding of secreted glycoproteins that pass through the ER. These substrates interact with ER-resident chaperones and chaperone-like lectins, whose activities in the glycan quality-control cycle are described. Once selected, the substrates are directed back to the cytoplasm where they are ubiquitylated and destroyed by the cytosolic proteasome via a process known as ER-associated degradation (ERAD) (Brodsky, 2013). Finally, Susan Michaelis and Eric Spear will focus on a quality-control pathway that exists in another cellular compartment, the cytoplasm. Even though a plethora of diseases arises from defective cytoplasmic proteins, the manner in which these proteins are recognized and destroyed has been enigmatic. However, a bounty of recent papers – many of which exploit the yeast model – have outlined the workings of the cytoplasmic quality-control system. As evident during ERAD, molecular chaperones play an important role during the selection of cytoplasmic substrates and, once selected, the proteins are also ubiquitylated and degraded by the proteasome.

We are confident that the readers of these expert reviews will develop a deeper appreciation of the many ways that cells cope with an onslaught of misfolded proteins, and the multiple effects on cellular health when these dedicated systems are unable to keep up with the insult that accompanies proteotoxic stress. We are also confident that the presented information will aid the search for new therapies to combat the consequences of protein conformational disorders. All of the articles published in the series will be collated into an online collection, which you can access via this dedicated page: http://dmm.biologists.org/site/protein-folding-disease.xhtml.
